# Genomic divergence in allopatric Northern Cardinals of the North American warm deserts is linked to behavioral differentiation

**DOI:** 10.1002/ece3.4596

**Published:** 2018-12-11

**Authors:** Kaiya L. Provost, William M. Mauck, Brian Tilston Smith

**Affiliations:** ^1^ Department of Ornithology American Museum of Natural History New York New York; ^2^ Department of Ecology, Evolution, and Environmental Biology Columbia University New York New York; ^3^ Richard Gilder Graduate School American Museum of Natural History New York New York; ^4^ Present address: New York Genome Center New York New York

**Keywords:** bird song, *Cardinalis cardinalis*, Cochise Filter Barrier, phylogeography, playback experiment, prezygotic isolation

## Abstract

Biogeographic barriers are considered important in initiating speciation through geographic isolation, but they rarely indiscriminately and completely reduce gene flow across entire communities. Explicitly demonstrating which factors are associated with gene‐flow levels across barriers would help elucidate how speciation is initiated and isolation maintained. Here, we investigated the association of behavioral isolation on population differentiation in Northern Cardinals *(Cardinalis cardinalis*) distributed across the Cochise Filter Barrier, a region of transitional habitat which separates the Sonoran and Chihuahuan deserts of North America. Using genomewide markers, we modeled demographic history by fitting the data to isolation and isolation‐with‐migration models. The best‐fit model indicated that desert populations diverged in the Pleistocene with low, historic, and asymmetric gene flow across the barrier. We then tested behavioral isolation using reciprocal call‐broadcast experiments to compare song recognition between deserts, controlling for song dialect changes within deserts. We found that male Northern Cardinals in both deserts were most aggressive to local songs and failed to recognize across‐barrier songs. A correlation of genomic differentiation and strong song discrimination is consistent with a model where speciation is initiated across a barrier and maintained by behavioral isolation.

## 1 INTRODUCTION

Populations are frequently separated by biogeographic barriers, the ecological and physical features in the landscape that prevent organisms with particular traits from dispersing across them (Coyne & Orr, 2004; Mayr, 1963; Simpson, 1940). Geographic isolation via barriers is thought to be the dominant mode of speciation (Coyne & Orr, 2004; but see Nosil, 2008; Pinho & Hey, 2010), but not all barriers indiscriminately and completely reduce gene flow across the entire community. Filter barriers, originally conceived as filter bridges (Simpson, 1940), are a specific type of feature that preferentially allow organisms with particular traits to pass through. Filters can be either abiotic or biotic in nature, occur on contemporary or historical scales, and can change over time, becoming more or less permissive (Lomolino, Riddle, & Brown, 2010). Certain populations exchange genes freely through these barriers while others show a complete cessation of gene flow, resulting in assemblages whose species have different patterns of genetic connectivity across the filter.

While the pattern of isolation by barriers is well known, the attributes that allow organisms to pass through biogeographic filters are comparatively understudied. The meeting of taxa at distinct geographic areas of secondary contact, or suture zones, has been documented across North America and other regions (Remington, 1968; Swenson, 2006; Swenson & Howard, 2004, 2005). While suture zones have shown that gene flow can occur in some taxa (Remington, 1968), they may also provide insight into why other taxa do not show introgression during secondary contact. Possible factors include variations in dispersal ability, differences in niche breadth or preferences, pre‐ and post‐zygotic reproductive barriers, or a combination of these. Understanding the importance of any of these mechanisms in preventing gene flow will clarify the link between the genetic structuring of populations and the subsequent initiation of speciation.

The Cochise Filter Barrier, which is a geological and ecological formation separating the Sonoran and Chihuahuan deserts in the southwestern United States and northern Mexico, is one example of the heterogeneous effects that filter barriers can have on the surrounding biota. The barrier formed during the uplift of the Sierra Madre Occidental and the Pliocene and Pleistocene glacial cycles (Morafka, 1977). Pleistocene glacial–interglacial cycles caused the Sonoran and Chihuahuan deserts to expand and contract repeatedly, alternately connecting the deserts via an arid corridor and separating the deserts with woodlands during colder glacial periods (Van Devender, 1990; Van Devender, Betancourt, & Wimberly, 1984). Under contemporary climatic conditions, the deserts are connected by a corridor of xerophytic vegetation (Holmgren, Norris, & Betancourt, 2007; Van Devender, 1990; Van Devender et al., 1984).

The genetic turnover of taxa between the Sonoran and Chihuahuan deserts generally occurs between the Baboquivari Mountains of Arizona and the Trans‐Pecos of Texas (108–112°W longitude; reviewed by Hafner & Riddle, 2011), but there is no narrow concordant transition zone across taxa (Pyron & Burbrink, 2010). Some species are genetically isolated across the Cochise Filter Barrier while others are unstructured or appear to maintain gene flow in birds (Riddle & Hafner, 2006; Zink, 2002; Zink, Kessen, Line, & Blackwell‐Rago, 2001) and other vertebrates (Castoe, Spencer, & Parkinson, 2007; Jaeger, Riddle, & Bradford, 2005; Mantooth, Hafner, Bryson, & Riddle, 2013; Myers, Hickerson, & Burbrink, 2017; Orange, Riddle, & Nickle, 1999; Pyron & Burbrink, 2009; Riddle & Hafner, 2006; Riddle, Hafner, & Alexander, 2000; Schield et al., 2017, 2015; Serb, Phillips, & Iverson, 2001); as such, the barrier is semipermeable to gene flow. For populations distributed across the Cochise Filter Barrier, it is unclear which mechanisms have facilitated or inhibited gene flow. To address one such mechanism by which barriers may prevent gene flow after isolation, we examined the role of behavior in a resident songbird, the Northern Cardinal (*Cardinalis cardinalis*).

In birds, a particularly salient prezygotic reproductive barrier comes in the form of song. Male songbirds sing to defend their territories and attract mates and behave aggressively toward conspecific songs and intruders (Catchpole & Slater, 1995; Gill & Lanyon, 1964). Juveniles learn their songs from nearby singing adults (e.g., Jenkins, 1978) and thus slight variations in dialect are retained in very localized areas as a consequence of low dispersal (Lanyon, 1979; Lemon, 1975; Marler, 1997; Slater, 1989). Females can discriminate against song types and dialects to choose mates (O'Loghlen & Rothstein, 1995; West, King, & Eastzer, 1981). Divergence in male traits may be associated with speciation, either directly through male–male competition or as mediated by female choice (Burdfield‐Steel & Shuker, 2018; Tinghitella et al., 2018; Uy, Irwin, & Webster, 2018). Assessing male–male competition is more tractable than female choice in experiments on wild birds because males more readily respond to experimental stimuli. Most studies done to date assumed that males and females behave similarly to each other in terms of response to male song (e.g., Derryberry, 2011; Dingle, Poelstra, Halfwerk, Brinkhuizen, & Slabbekoorn, 2010). The few studies that have assessed both sexes have found no evidence that males are more discriminating than females, supporting such an assumption (Uy et al., 2018). Thus, to obtain larger samples sizes in order to tease apart the effects of ancestry and geography on song discrimination, we focused on male responses in this study.

If males on either side of a barrier sing different songs, females may not recognize a novel song as a reproductive signal, reducing interactions between populations and preventing successful gene flow (Hunt, Breuker, Sadowski, & Moore, 2009; Lipshutz, 2017b; Searcy & Andersson, 1986). Populations would thus become isolated, and differentiation would be maintained by behavioral isolation. Like other songbirds, Northern Cardinal males are generally less aggressive in response to unfamiliar songs, and the species is sensitive to dialect changes that can occur over tens of kilometers, responding with decreased levels of aggression to more distant dialects (Anderson & Conner, 1985; Lemon, 1966, 1974). Given this sensitivity, Northern Cardinals are likely to use song recognition as a means of species recognition, making them a candidate for investigating the relationship between genetic connectivity and song divergence. At present, however, the impact of song variation across the deserts has not been examined with respect to the impact of dialect changes due to geography. Such an assessment would disentangle the roles of dialect changes due to geographic distance versus dialect changes due to allopatry across a barrier, sexual selection, or reproductive character displacement.

The Northern Cardinals in the Sonoran and Chihuahuan deserts are a tractable model for testing these changes as they are currently allopatric without a known contact zone. Northern Cardinals have a fragmented distribution across the Cochise Filter Barrier, being separated by a gap of ~200 km that corresponds to the elevational and environmental change of the Cochise Filter Barrier. Due to this, there should be no contemporary impact on the dialects in this region either from song learning or from reinforcement. Thus, this system allows for the study of the early stages of speciation that are unbiased by ongoing secondary contact, and our approach controls for dialect changes over large distances without relying on contact zones as proxies for connectivity.

Prior work has shown that this species shows phenotypic (Ridgway, 1901) and genetic (Smith & Klicka, 2013; Smith et al., 2011) differentiation across the barrier. At present, however, the amount of gene flow that occurs across the barrier, either currently or historically, has not been quantified. There are multiple potential factors that could have led to the separation of these lineages. From a pure dispersal standpoint, Northern Cardinals should readily be able to cross this region over evolutionary time assuming no environmental or behavioral barriers. The Northern Cardinal has undergone dramatic contemporary range expansions into the northern United States and Canada and there are numerous records of individuals that have dispersed a similar distance or greater across unsuitable habitat (Halkin & Linville, 1999). Further, vagrant Northern Cardinals are regularly observed well outside their species’ resident distribution (Sullivan et al., 2009). Reconstruction of Pleistocene species distribution models also indicates that there were more suitable climatic conditions across the Cochise Filter Barrier during glacial cycles (Smith et al., 2011). Thus, it is unlikely that the observed differentiation across the Cochise Filter Barrier is due solely to limitations on this species’ dispersal capabilities. Instead, it seems more likely that other traits have impacted divergence in this species.

How focal populations respond to vocal dialects is expected to be linked to the magnitude and direction of gene flow across the Cochise Filter Barrier. Individuals who successfully migrate should exchange their genetic material and dialect with the local population. When no gene flow occurs across the barrier (i.e., pure isolation), both populations should respond aggressively to their own dialect and should ignore the other desert's dialect. Likewise, if gene flow occurs equally across the barrier in both directions (i.e., isolation with symmetric gene flow), then focal populations should respond equally aggressively to both their own dialect and to the other desert population's dialect. When gene flow is biased in one direction (i.e., isolation with asymmetric or unidirectional gene flow), one population is exposed to both dialects while the other is exposed only to their own. Because of this, the population receiving more migrants should respond aggressively to both dialects. However, the population that receives fewer migrants should respond less aggressively to the foreign dialect, or even ignore it. Populations that have come into secondary contact are predicted to show equal aggression to dialects if focal populations are tested within the contact zone, and ignore foreign songs outside of the secondary contact zone.

**Figure 1:**
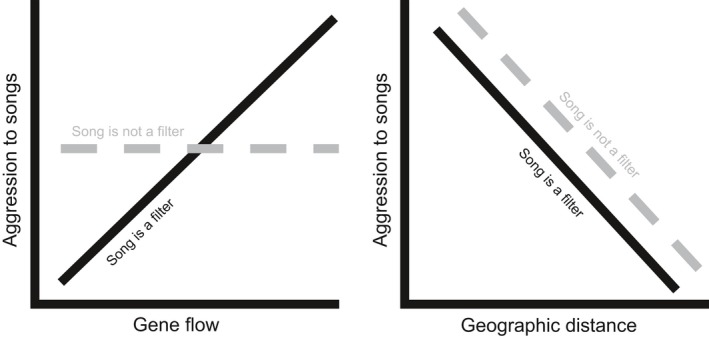
Illustration of hypothesized relationships between gene flow and song (left panel) and geographic distance and song (right panel). If song acts as a reproductive filter (solid black line), male aggression to non‐local songs should be high if gene flow between their populations is high (and thus if genetic isolation is low). However, if song discrimination does not act as a reproductive filter (dashed gray line), there should be no correlation between gene flow among populations (or genetic isolation) and aggression. Irrespective of whether song acts as a filter, populations that are at larger geographic distances should show lower aggression to songs due to dialect changes

Here, we tease apart these complex scenarios by integrating demographic modeling of genomewide genetic variation and field‐based experiments to test how a barrier regulates speciation. First, we characterized population structure and fit genomic data to pure isolation and isolation‐with‐migration models. From these analyses, we inferred the depth of divergence and timing of gene flow across the barrier. Second, we performed call‐broadcast experiments in each desert to assess male aggression to local and non‐local songs. If song discrimination is a reproductive filter, then we predict that isolation and the extent of gene flow will be correlated with male aggression to non‐local songs (Figure 1). By combining genomic estimates of isolation and introgression with responses of wild birds to song differences involved in mate choice, we explored whether behavioral isolation helps regulate gene flow across filter barriers.

## 2 MATERIALS AND METHODS

### 2.1 Collection of genomic data

**Figure 2:**
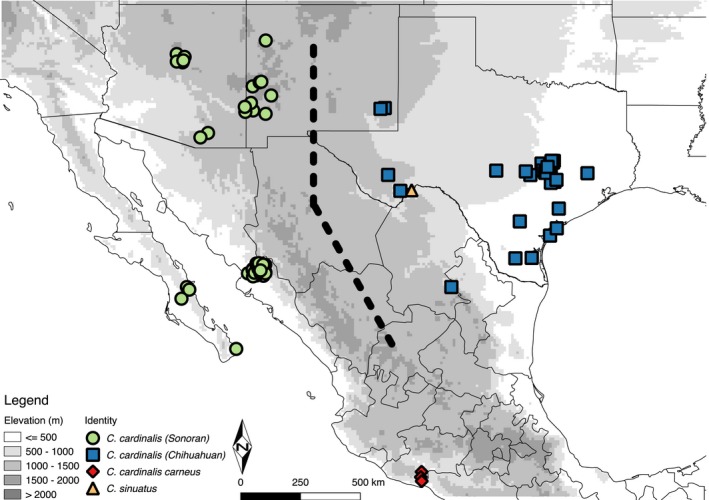
Location of vouchered *Cardinalis cardinalis* genetic samples. Points are jittered slightly to avoid overlap. Note that Chihuahuan group includes all samples east of the Cochise Filter Barrier, including individuals collected outside the Chihuahuan Desert proper. Black dotted line shows the approximate region of the Cochise Filter Barrier

We sequenced genomewide markers from vouchered genetic samples collected east and west of the Cochise Filter Barrier (Figure 2; Table A1). Northern Cardinals are sparse in the region of the barrier itself and as such we lack sampling there (Sullivan et al., 2009). All of the western samples occur in the Sonoran Desert (*N* = 54) while the eastern samples include the Chihuahuan Desert and adjacent areas in New Mexico and Texas (*N* = 31). For simplicity, we designated western individuals the Sonoran population and eastern individuals the Chihuahuan population, though they include individuals outside of the deserts proper. These correspond to the *igneus* and *cardinalis* lineages identified in previous work (Ridgway, 1901; Smith & Klicka, 2013; Smith et al., 2011). We also included three individuals of *C. cardinalis carneus* from the Pacific Coast of Mexico and one of *C. sinuatus* as outgroups.

We used a Qiagen DNeasy blood and tissue kit to isolate pure genomic DNA for later sequencing following the built‐in protocol with minor modifications. Briefly, in the final elution step, we added two elutions of 200 μl of water to ensure all DNA had been removed from the filter, then concentrated the elution into a volume of 32 μl. We quantified the concentration of the extracted DNA using Qubit Fluorometric Quantitation (Life Technologies). Double‐digest restriction‐site‐associated DNA sequence (ddRAD) libraries were prepared and sequenced at the University of Texas Austin Genomic Sequencing and Analysis Facility using a protocol modified from Peterson, Weber, Kay, Fisher, and Hoekstra (2012) (See Appendix 1 for details). The ddRAD libraries were sequenced on a single lane of an Illumina HiSeq 4000 PE 2x150, producing paired‐end reads of approximately 200–300 base pairs. We processed fastq files using PyRAD version 3.0.66 (Eaton, 2014; Settings: Mindepth 6, NQual 4, Wclust 0.85, Datatype pairddrad, MinCov 4, MaxSH 3, maxM 0, filter NQual+adapters, maxH 10, trim overhang 2,2; see Appendix 1 for more details). Data were processed for all individuals (including outgroups) as well as for only Sonoran and Chihuahuan individuals.

We characterized genetic structure with a STRUCTURE analysis (Hubisz, Falush, Stephens, & Pritchard, 2009; Pritchard, Stephens, & Donnelly, 2000), running five runs each of clusters *K* = 1 to *K* = 5 for 100,000 generations of burn‐in and 500,000 generations of run time. Note that for the ingroup‐only dataset, we ran 10 runs each instead of five runs each. This technique assigns individuals to a specified number of *K*‐clusters and outputs the log‐likelihood of the *K*‐cluster in question. These runs were automated with the program StrAuto 0.3.1 (Chhatre & Emerson, 2017). We evaluated the best *K* value using both the Evanno, Regnaut, and Goudet (2005) method and the Puechmaille (2016) method, as implemented in StructureSelector (Li & Liu, 2018). For the Puechmaille (2016) method, we used a mean membership threshold value of 0.5 after testing values between 0.5 and 0.9 (see Appendix 1 for those results). To visualize STRUCTURE data, we used STRUCTURE Harvester (Earl & vonHoldt, 2012), CLUMPP 1.1.2 (Jakobsson & Rosenberg, 2007) and DISTRUCT (Rosenberg, 2004), as well as custom scripts in R. The STRUCTURE analyses were run on a dataset excluding the outgroups (see Appendix 1 for the dataset including the outgroups). Additionally, to supplement the STRUCTURE results, we calculated Nei's *G*_ST_ (Nei, 1973, 1987 ) and Hedrick's *G*'_ST_ (Hedrick, 2005) from the single nucleotide polymorphism (SNPs) we extracted via the package vcfR (Knaus & Grünwald, 2017).

### 2.2 Demographic modeling

**Figure A2:**
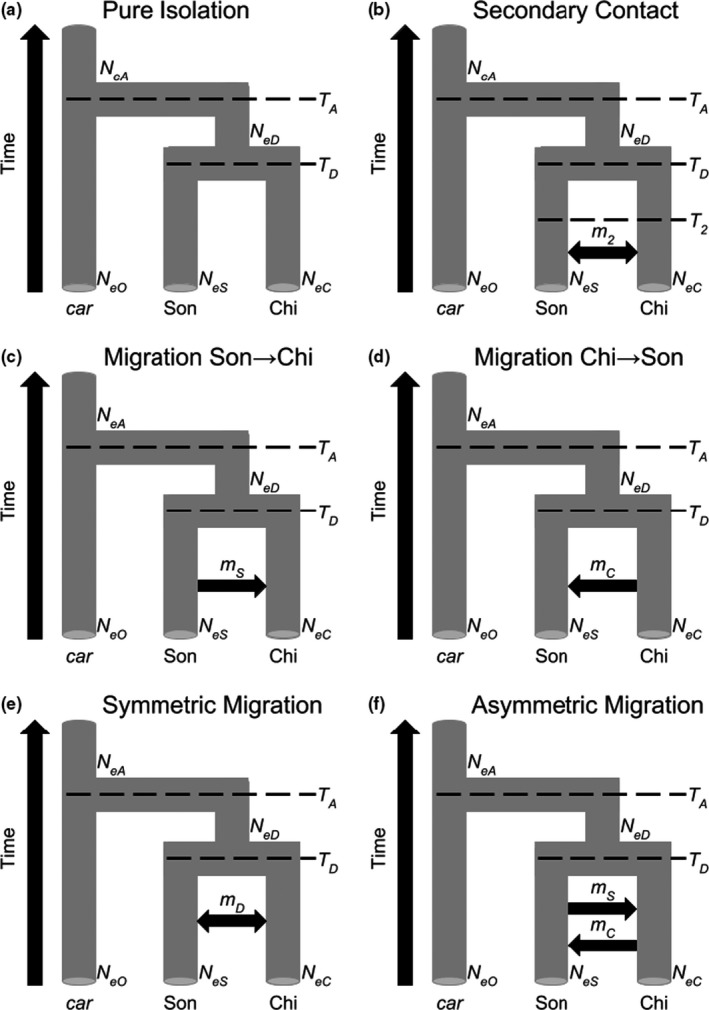
Demographic models used in fastsimcoal2. Panels a–f describe six demographic scenarios. Shown are effective population sizes in haploid individuals (*N*_e_), migration rate between deserts in individuals per year (*m*), and time of divergence or secondary contact in years (*T*). Subscripts designate the following: *A*: ancestor to *C. cardinalis* complex; *D*: ancestor to Sonoran–Chihuahuan desert groups; *S*: Sonoran group (Son); O: outgroup *C. c. carneus* (*car*); *C*: Chihuahuan group (Chi). Note that in all cases, *T_A_* is fixed at 2 million years

We modeled demographic history (*N*_E_ = effective population size, *T* = time of divergence, and *m* = gene flow between populations) and performed model selection using fastsimcoal2 version 2.52.21 (Excoffier & Foll, 2011; Excoffier, Dupanloup, Huerta‐Sánchez, & Foll, 2013). Using SNP data in variant call format (VCF) from PyRAD, we generated an unfolded joint site frequency spectrum (SFS) using ∂a∂i (Gutenkunst, Hernandez, Williamson, & Bustamante, 2009) and simulated from it in fastsimcoal2, using a custom script available on GitHub (available at https://github.com/isaacovercast/easysfs). We projected the SFS down to a smaller number of samples (10 Sonoran × 10 Chihuahuan × 2 *C. c. carneus*), averaging over missing data for each population. We then used the SFS to simulate demographic histories under multiple models, testing the fit of the simulations to our empirical SFS data. We then used the SFS to simulate demographic histories under multiple models, testing the fit of the simulations to our empirical SFS data, assuming a mutation rate of 2.21 × 10^−9^ mutations per site per year (*µ*, used to estimate *N*_E_ from *θ* = 4 *N*_E_ *µ*; Nam et al., 2010) and generation time of 1 year based on the year of first breeding of the species (Halkin & Linville, 1999). We tested six demographic models (Figure A2) representing isolation with or without gene flow between the Sonoran and Chihuahuan deserts, where isolation refers to allopatric populations: (a) pure isolation, (b) isolation with symmetric migration, (c) isolation with asymmetric migration, (d) isolation with migration from Sonoran to Chihuahuan only, (e) isolation with migration from Chihuahuan to Sonoran only, and (f) isolation with secondary contact that allowed gene flow since the last glacial maximum to the present. The models used three populations: the Sonoran group, Chihuahuan group, and the outgroup, *C. c. carneus*.

For each model, we ran 25 iterations of 100,000 simulations on 100 parameter sets, selected the iteration with the highest estimated maximum likelihood, and chose the best model by comparing Akaike information criterion (AIC) scores. We considered a model that improved the AIC score (∆AIC) by 2.0 to be a significant improvement, with an improvement of 10.0 or more highly significant. We then chose the best model and ran 100 bootstraps (100,000 simulations of 100 parameter sets) to calculate mean and 95% confidence interval estimates for effective population sizes, gene flow rates, time of divergence, and time of secondary contact.

The distribution and sampling ranges for model parameters were as follows (distribution; range): effective population sizes (log‐uniform distribution; 50,000–1,000,000 haploid individuals), migration (log‐uniform distribution; 0.001–20 individuals per generation), the Sonoran–Chihuahuan divergence time (uniform distribution; 500,000–1,000,000 years), and the time of secondary contact (uniform distribution; 0–21,000 years). Note that in fastsimcoal2, upper bounds on the ranges are soft boundaries and parameter values higher (but not lower) than these bounds can be estimated. Finally, we fixed the split between the Sonoran–Chihuahuan clade and *C. c. carneus* to 2,000,000 years (Smith et al., 2011) to calibrate the parameters into absolute values.

### 2.3 Testing behavioral isolation across the Cochise Filter Barrier

**Figure 3:**
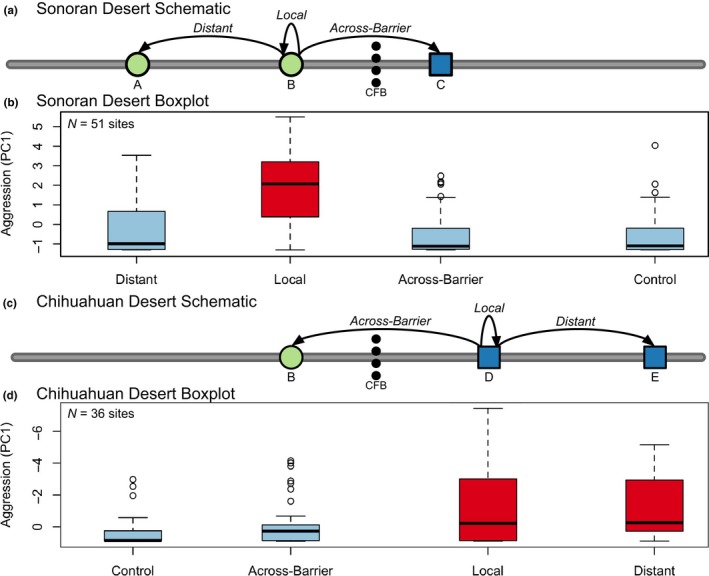
Male Northern Cardinals in both deserts were most aggressive to Local songs. Panels combine schematic of playback experiment (a, c) with boxplots showing mean aggression scores (b, d). Sonoran Desert results shown in Panels a and b. Chihuahuan Desert results show in Panels c and d. Panels a and c: Circles show localities within the Sonoran Desert population; squares show localities within the Chihuahuan Desert population. Distances between localities represent longitudinal distance. Arrows point from focal population of each experiment to location of Local, Distant, and Across‐Barrier recording treatments (Control treatment is omitted). Letters indicate locality names: A) Western Arizona: Bill Williams River National Wildlife Refuge, AZ, Santa Maria River National Wildlife Refuge, AZ, and Wenden, AZ; B) Portal, AZ; C) Rattlesnake Springs Reserve, NM; D) Big Bend National Park, TX; E) Eastern Texas: Balcones Canyonlands National Wildlife Refuge, TX, and Austin, TX. Panels b and d: Boxplots show mean aggression scores (PC1, *y*‐axis) for each song treatment (*x*‐axis). Values at the top of the graph indicate higher aggression. Sites without detections were excluded. Red bars are statistically significantly different from blue bars. Note that because Chihuahuan PC1 is negatively correlated with aggression, Chihuahuan *y*‐axis is inverted with more negative PC1 values toward top of graph (Sonoran PC1 is positively correlated with aggression)

We used a playback (call‐broadcast) experiment to examine the response of males to simulated intruders (Peters, Searcy, & Marler, 1980; Derryberry, 2007; Derryberry, 2011; see Appendix 2 for details of protocol). These experiments assessed aggression toward a treatment, that is, songs from one of three geographic areas and a control. Playbacks were in accordance with Columbia University's Institutional Animal Care and Use Committee (approved as Protocol AAAM5551). We performed experiments on both Sonoran and Chihuahuan individuals in mesquite scrub habitat. Sonoran playback sites were near Portal, AZ, and Chihuahuan playback sites were in Big Bend National Park, TX. For each desert experiment, we categorized recordings into one of four treatments: Local, Distant, Across‐Barrier, and Control (Figure 3). Local recordings came from the same population whose responses we measured (i.e., the focal population). Distant recordings came from the same desert as the focal population, but from a large geographic distance (~450–625 km). Across‐Barrier recordings came from the other desert lineage, which were necessarily at a large geographic distance. Distant songs and Across‐Barrier songs should have been novel to the Local population for both desert populations (Lemon, 1975). Control recordings were that of a Cactus Wren (*Campylorhynchus brunneicapillus*), which is a distantly related bird common in both deserts. We chose these recordings to compare the effects of distance and presumed genetic relatedness on a population's response to a recording.

Some recordings were downloaded from Xeno‐Canto (xeno‐canto.org), an online open‐access birdsong repository (XC141499, XC141500, XC240937, XC233123, XC233122, XC211632, XC211631). All other recordings were made by the authors using a Sennheiser ME66 shotgun microphone connected to a Roland R‐26 portable recorder at a sample rate of 96 kHz and a sample size of 24 bits. For each recording, we created a stimulus set of approximately 3‐min duration (range: 2:59–3:10 min). Each stimulus set included three 50‐s bouts of song, with 10‐s periods of silence following each bout. We added these silent periods to mimic multiple singing bouts as Northern Cardinals in these areas rarely sang continuously for 3 min (see Appendix 2). We used 5–7 stimulus sets for each treatment except for the Control, in which we had one single Cactus Wren recording. We used an HTC6500LVW cell phone and an HMDX Jam Bluetooth speaker (model HX‐P230GRA) with a ~1‐m‐long 3.5‐mm audio cable to play the stimulus sets. We placed the speaker on a tripod ~1 m off the ground. Stimulus sets were standardized to 80–85 dB as measured 1 m away from the speaker. We broadcasted multiple stimulus sets per recording locality (or Treatment) to control for pseudoreplication (Kroodsma, 1989). The Control stimulus set was played at every site (*N* = 67 Sonoran, 61 Chihuahuan). We played the Sonoran stimulus sets 11–15 times each, except for two Across‐Barrier stimulus sets which were played once and twice, respectively. The Chihuahuan stimulus sets were played 9–17 times each.

### 2.4 Behavioral study sites

To choose sites for behavioral experiments, we placed GPS points in habitat known to have territorial males. We did not verify that points had resident males before beginning playback experiments on those sites. When males were found, we did not mark individuals but instead assumed that males found on territories were the territory holders. Sonoran Northern Cardinal territories in Pima County, AZ, average 1.56 ha (Gould, 1961) while Chihuahuan territories in Nacogdoches County, TX, have a mean ± standard deviation of 0.64 ± 0.14 ha (Conner, Anderson, & Dickson, 1986). Territory sizes range within the species from 0.21 to 2.60 ha (Halkin & Linville, 1999) with size partially dependent on foliage (Conner et al., 1986). Assuming circular territory shape, this species forms territories with radii averaging 70.5 m in the Sonoran Desert and 45.1 m in the Chihuahuan Desert (range: 25.8–91.0 m). We assumed that distances between sites were sufficient to minimize territorial overlap and that each site comprised a single territory. Distances between Sonoran nearest‐neighbor sites ranged from 38 to 205 m. Distances between Chihuahuan nearest‐neighbors were 92–204 m. We generated transects of 6–13 sites each whose playbacks we always completed in the same order, barring dangerous conditions such as sudden inclement weather (see Tables A2, A3). We did this to minimize time‐of‐day and time‐of‐year effects within sites. Sites within transects were at least 90 m apart to minimize repeat testing of the same territories.

Playback experiments were done during the breeding season of 2015 for Sonoran birds and 2016 for Chihuahuan birds. We performed all playbacks at each site at nearly the same time each day, completing all trials within 9 days of initiating experiments at a site. Males had at least ~24 hr between playbacks to return to a non‐disturbed state. Two Chihuahuan sites form exceptions to these rules as they had multiple playbacks per day, were done substantially later in the day, and with less than an hour between playbacks.

We conducted four playbacks at each site, randomly selecting one stimulus set from each of the four Treatment localities (Local, Distant, Across‐Barrier, and Control). We observed the site for 3 min before playback began (“Pre‐Playback” period). We then played the 3‐min stimulus set (“Playback” period) and continued to observe the male during a silent post‐playback period of 3 min (“Post‐Playback” period). In most analyses, we combined the Playback and Post‐Playback periods a posteriori into a “Response” period. During each period, we recorded multiple aggression measures: (a) the number of flights >1 m, or “Flybys,” within the site; (b) the presence of alarm calls, or “Calls,” within the site; (c) the number of male songs produced within the site, or “Close Songs”; (d) the number of male songs produced outside the site, or “Far Songs”; and (e) the distance of males from the speaker recorded every 10‐s, or “Distance by Time.” Distance to playback equipment is a known proxy for avian aggression and mating signal recognition (Searcy, Anderson, & Nowicki, 2006). We categorized distances into distance bins (0–1 m, 1–2 m, 2–4 m, 4–8 m, 8–16 m, 16–24 m, and >24 m) using markers placed 8 m and 16 m from the speaker. We chose these bins as they were easily estimated by observers while also accurately capturing distance variation of male Northern Cardinals during preliminary trials. We localized birds that disappeared from sight to their nearest distance bin by sound if we could track them without ambiguity. If the localization was ambiguous, we classified the individual as >24 m (out of the site). From these distance records, we computed the minimum distance to speaker, or “Closest Distance,” for each period.

The five aggression measures were reduced to a single composite aggression measure via principal components analysis (PCA). We calculated the principal components using Response period (Playback and Post‐Playback combined) data, then used the resulting loadings to calculate a posteriori the first principal component, PC1, for the Pre‐Playback period, or “Pre‐Treatment Aggression” measure. We used generalized linear mixed models (GLMMs) to determine whether there was a correlation between aggression (PC1) and Treatment. We set Treatment and Pre‐Treatment Aggression as fixed effects and included the random effects of site and stimulus set in our models to control for individual site differences and differential responses to the various stimulus sets from the same locality. Each playback (unique combination of site and stimulus set) was used as a replicate. To evaluate the importance of Treatment as a predictor, we compared the outputs of (a) a model containing all variables and (b) a model with all variables except for Treatment. We chose the model with the smallest AIC corrected for small sample size (AIC_C_) value as the best model, evaluated the significance of Treatment and Pre‐Treatment Aggression predictors using a Wald chi‐square test, and assessed model fit by calculating adjusted *R* ^2^ values.

## 3 RESULTS

### 3.1 Raw genomic results from ddRAD pipeline

We collected 67,191 raw loci from our ddRAD pipeline. After filtering for paralogs, this was reduced to 33,626 processed loci, from which we extracted 28,798 unlinked SNPs out of a total of 361,011 variable sites and 148,370 parsimony‐informative sites. Two individuals had high amounts of missing data which led to spurious assignment to groups in preliminary analyses; as such, these individuals were removed from further analyses (see Appendix 1). All other individuals had between 3,353–9,752 loci associated with them (mean ± standard deviation 7,000 ± 1,490, median 6,917).

### 3.2 Population structure and demography

**Figure 4:**
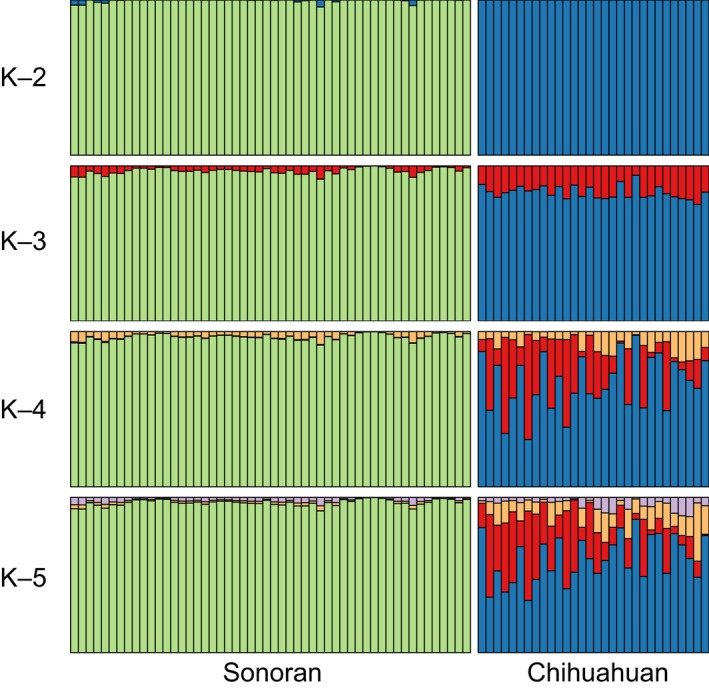
Individuals east and west of the Cochise Filter Barrier were consistently assigned to different populations. Results from STRUCTURE runs *K* = 2–5 are presented. Each vertical bar represents an individual bird, with the proportion of each color indicating assignment to that population. See Figure A1 for preliminary results

STRUCTURE analyses on Sonoran and Chihuahuan individuals showed strong population assignments, with Sonoran individuals always assigned to one cluster and Chihuahuan individuals never assigned to that cluster (Figure 4). The highest log‐likelihood support values were for *K* = 2 (mean ±standard deviation −181,543 ± 56) and *K* = 3 (−186,576 ± 131) populations. Comparisons of ∆*K* (Evanno et al., 2005) show highest support for *K* = 3. However, when *K* = 3, the third cluster never achieves more than 25% assignment probability in any individual. Using the methodology suggested by Puechmaille (2016) implemented in Structure Selector (Li & Liu, 2018), the highest support for all metrics is *K* = 2.

**Figure 5:**
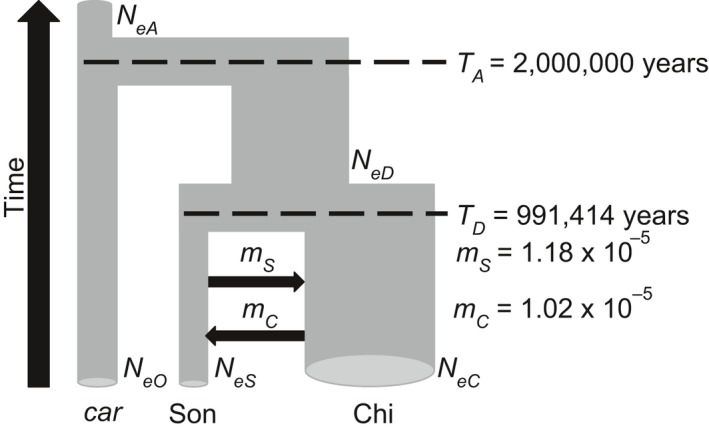
The best‐fit demographic model was isolation with asymmetric migration. Shown are mean effective population sizes in haploid individuals (*N_e_*), mean migration rate between deserts in individuals per year (*m*), and mean time of divergence in years (*T*). Width of tubes is proportional to mean *N_e_*. Subscripts designate the following: *A*: ancestor to *Cardinalis cardinalis* complex; *D*: ancestor to Sonoran–Chihuahuan desert groups; *S*: Sonoran group (Son); O: outgroup *C. c. carneus* (*car*); *C*: Chihuahuan group (Chi). Mean (95% CI) for each: *N*_eA_ 181,138 (125,535–269,034), *N*_eD_ 776,869 (537,911–722,740), *N*_eO_ 212,169 (147,239–304,927),* N*_eS_ 153,451 (103,168–233,928), *N*_eC_ 706,389 (607,515–792,290), *T*_D_ 991,414 (912,448–1,079,034), *m*_S_ 1.18 × 10^−5^ (2.11e‐06–3.60 × 10^−5^), *m*_C_ 1.02 × 10^−5^ (7.24 × 10^−6^–1.38 × 10^−5^). Note that *T*_A_ is fixed at 2 million years. See Figure A2 for all models tested. Image modified from Carstens et al. (2013)

Our demographic analyses with fastsimcoal2 found that a model with asymmetric gene flow (Figure 5) was best supported over all other models (AIC = 12,876.398, best ΔAIC for all other models range 3.7–1,129.3; Table A4). After bootstrapping the model, we dated the divergence between the Sonoran and Chihuahuan population to be a mean of 991,414 years (95% CI 912,448–1,079,034). The Sonoran effective population size (mean 153,451; 95% CI 103,168–233,928) was smaller than the Chihuahuan (mean 706,389; 95% CI 607,515–792,290); this was true across all models, including ones with lower likelihood scores (Table A4). Though the models with gene flow received higher support than models without (Table A4), the actual estimated gene flow rates across the barrier were minute for both the Sonoran‐to‐Chihuahuan (mean 1.18 × 10^−5^; 95% CI 2.11 × 10^−6^–3.60 × 10^−5^) and Chihuahuan‐to‐Sonoran routes (mean 1.02 × 10^−5^; 95% CI 7.24 × 10^−6^–1.38 × 10^−5^).

### 3.3 Levels of aggression to playbacks from different localities

We ran playback experiments at 67 and 61 sites in the Sonoran and Chihuahuan deserts, respectively, resulting in 512 total playback trials (four playbacks per site). Of these, we did not detect males at 16 Sonoran and 25 Chihuahuan sites. These sites were removed, leaving 51 Sonoran and 36 Chihuahuan sites, for 348 total playbacks. When we performed our PCA on the aggression data, the first principal component (PC1, aggression) explained 58.2% of the variation in the Response period data for the Sonoran individuals and explained 52.4% of variation in the Response period for the Chihuahuan individuals. Sonoran PC1 ranged from −1.30–5.95, with more positive values indicating higher aggression. Chihuahuan PC1 ranged from −7.44–0.91, with more negative values indicating higher aggression.

We compared the effects of GLMMs with and without recording locality (Treatment) as a predictor and found that Treatment had a significant effect on male aggression in both deserts (Table 1). For playbacks on Sonoran individuals, the model with Treatment was a better fit to the Aggression data than the model without (∆AIC_C_ = 9.97), though both models had equivalent adjusted *R* ^2^ values (0.90). Wald tests indicated that both Treatment and Pre‐Treatment Aggression were significant factors in the full model (Treatment *p*‐value <0.001; Pre‐Treatment Aggression *p*‐value < 0.001). Sonoran males were significantly more aggressive to Local stimulus sets than to those from any other location (Local mean PC1 = 1.52; Distant mean PC1 = −0.36; Across‐Barrier PC1 = −0.54; Control mean PC1 = −0.62; all *p*‐values <0.001; Figure 3). By contrast, there were no significant differences in aggression across Distant, Across‐Barrier, or Control stimulus sets (all *p*‐values ≥ 0.35).

**Table 1:** Models including treatment locality explain responses to song dialects better than models without

	Sonoran experiment		Chihuahuan experiment	
	Null model^a^	Full model^b^	Null model^a^	Full model^b^
AIC_C_ (∆AIC_C_)	588.7 (9.96)	579.01 (0.00)	352.87 (4.79)	348.08 (0.00)
adj*R* ^2^ (*R* ^2^)	0.90 (0.90)	0.90 (0.90)	0.70 (0.70)	0.66 (0.67)
Site variance	0.04	0.04	0.00	0.00
Stimulus set variance	0.14	0.03	0.40	0.07
Residual variance	0.34	0.33	1.15	1.26
Intercept *α* ± *SE*	1.02 ± 0.13	0.60 ± 0.21	−0.56 ± 0.20	−0.61 ± 0.33
Intercept *T* (*p*‐val)	7.69 (1.47 × 10^−^^14^)	2.79 (0.0054)	2.84 (0.0045)	−1.84 (0.065)
Pre‐Agg *β* ± *SE*	0.39 ± 0.08	0.39 ± 0.08	−0.70 ± 0.12	−0.71 ± 0.12
Pre‐Agg *T* (*p*‐val)	4.93 (8.15 × 10^−^^7^)	5.06 (4.36 × 10^−^^7^)	5.88 (4.05 × 10^−^^9^)	−5.81 (6.40 × 10^−^^9^)
Con versus Acr *β* ± *SE*	N/A	0.10 ± 0.23	N/A	0.89 ± 0.40
Con versus Acr T (*p*‐val)	N/A	0.45 (0.65)	N/A	2.22 (0.027)
Con versus Dis *β* ± *SE*	N/A	0.18 ± 024	N/A	1.57 ± 0.40
Con versus Dis T (*p*‐val)	N/A	0.78 (0.44)	N/A	3.92 (9.00 × 10^−^^5^)
Con versus Loc *β* ± *SE*	N/A	1.09 ± 0.24	N/A	1.60 ± 0.40
Con versus Loc T (*p*‐val)	N/A	4.65 (3.27 × 10^−^^6^)	N/A	4.00 (6.14 × 10^−^^5^)
Acr versus Dis *β* ± *SE*	N/A	0.078 ± 0.16	N/A	0.67 ± 0.32
Acr versus Dis T (p‐val)	N/A	0.48 (0.63)	N/A	2.10 (0.036)
Acr versus Loc *β* ± *SE*	N/A	0.99 ± 0.16	N/A	0.71 + 0.32
Acr versus Loc T (*p*‐val)	N/A	6.07 (1.26 × 10^−^^9^)	N/A	2.21 (0.027)
Dis versus Loc *β* ± *SE*	N/A	0.91 ± 0.16	N/A	0.04 ± 0.32
Dis versus Loc T (*p*‐val)	N/A	5.53 (3.13 × 10^−^^8^)	N/A	0.12 (0.91)

Note

Model parameter results from generalized linear mixed models for both Sonoran and Chihuahuan experiments.

Pre‐Agg: Pre‐Treatment Aggression; *SE*, standard error; Treatment localities are as follows: Con: Control; Acr: Across‐Barrier; Dis: Distant, Loc: Local.

a Null model formula: Aggression ~ Pre‐Treatment Aggression + 1|Stimulus Set + 1|Site. Pre‐Treatment Aggression is a fixed effect, while 1|Stimulus Set and 1|Site are random effects.

b Full model formula: Aggression ~Treatment + Pre‐Treatment Aggression + 1|Stimulus Set +1|Site. Pre‐Treatment Aggression and Treatment are fixed effects, while 1|Stimulus Set and 1|Site are random effects.

For playbacks on Chihuahuan individuals (Table 1), AIC_C_ scores supported the model with Treatment over the model without (∆AIC_C_ = 4.79), though the model without Treatment had trivially greater explanatory power according to adjusted *R* ^2^ (0.70 vs. 0.66). Nevertheless, Wald tests indicated that both Treatment and Pre‐Treatment Aggression were significant factors in the full model (Treatment *p*‐value = 0.027; Pre‐Treatment Aggression *p*‐value < 0.001). Chihuahuan males were significantly more aggressive to Local and Distant stimulus sets than to Across‐Barrier or Control stimulus (Local mean PC1 = −0.42; Distant mean PC1 = −0.32; Across‐Barrier mean PC1 = 0.23; Control mean PC1 = 0.52; all *p*‐values ≤0.05; Figure 3). Local and Distant were not statistically different, and neither were Across‐Barrier and Control (all *p*‐values ≥ 0.5).

**Figure 6:**
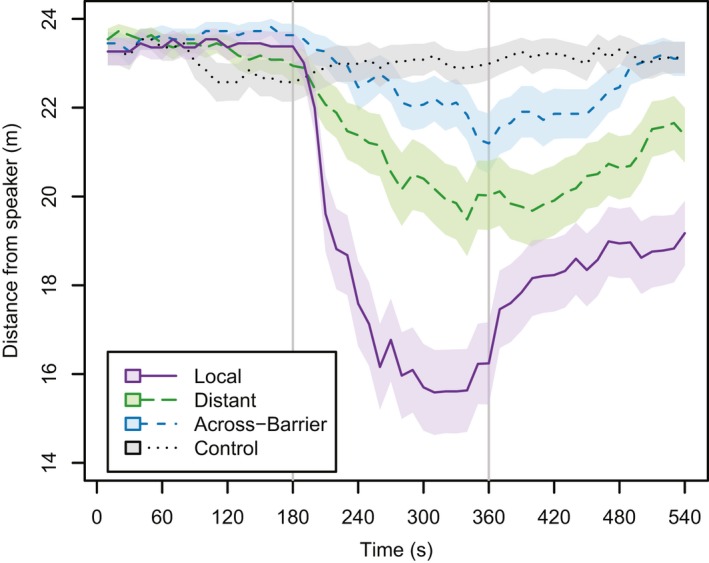
Individuals increased aggression when playbacks begin. Mean distance from speaker (m) over time for each treatment, with smaller distance values indicating more aggression. Data from both deserts were combined (*N* = 87 sites). Lines indicate mean distance values across all treatments, with shading around lines indicating one standard error around points. Solid purple line indicates Local treatment, large‐dashed green line indicates Distant treatment, medium‐dashed blue line indicates Across‐Barrier treatment, and dotted gray line indicates Control treatment. Vertical lines separate Pre‐Playback (left), Playback (middle), and Post‐Playback (right) periods

Both Sonoran and Chihuahuan birds shifted their behavioral response across the experimental periods (i.e., Pre‐Playback, Playback, and Post‐Playback) (Figure 6). Sonoran individuals hearing the Local treatment, and Chihuahuan individuals hearing either the Local or Distant treatment, were substantially closer to the speaker during the Playback and Post‐Playback periods compared to the Pre‐Playback periods. There were no significant differences between Pre‐Treatment Aggression values for Treatment, Population Tested, or their interaction (all *p*‐values ≥ 0.17). In contrast, all values were highly significant when analyzing PC1 values (all *p*‐values < 0.001). The significant interaction indicates that there were significant differences between the deserts in the response to a treatment. Sonoran individuals are significantly more likely to be aggressive to Local songs than Chihuahuan individuals (*p* < 0.001), though there are not significant differences for the other three treatments (all *p*‐values ≥ 0.17). Across all of these analyses, male Northern Cardinals in both deserts show minimal aggression toward Across‐Barrier songs, treating the songs of a foreign desert as heterospecific.

## 4 DISCUSSION

We found that the Northern Cardinal had low gene flow and strong male song discrimination across the Cochise Filter Barrier. Further, we showed that song discrimination between deserts is greater than song discrimination within deserts, indicating that these birds exhibit divergence in song beyond what would be expected through dialect changes alone. This strong discrimination against songs from an allopatric population may mediate the degree of gene flow permeability among deserts. Analogous studies to this one frequently examine differences between groups that are adjacent to one another (e.g., Dingle et al., 2010; Lipshutz, Overcast, Hickerson, Brumfield, & Derryberry, 2017), allowing for tests along axes of sympatric versus parapatric or within versus outside a hybrid zone. However, Northern Cardinals have no known contemporary contact zone, and we found no support for ongoing gene flow, across the Cochise Filter Barrier. The two allopatric populations have higher connectivity within than between deserts, though the latter could still have been historically high in short bursts. This is in contrast to a hybrid zone system in which connectivity is relatively high in the localized area of contact. Instead of focusing on what occurs during secondary contact, studying allopatric populations allowed us to show how behavioral divergence evolves in isolation and impacts genomic divergence.

### 4.1 Potential modes of speciation across the Cochise Filter Barrier

We found that song discrimination and gene flow between allopatric populations were correlated. One potential relationship between these two factors is that song could act as a driver of divergence, with or without allopatry (Uy et al., 2018). The song differences observed in this study may have directly caused genomic divergence through assortative mating during periods of contact (Bensch, Hasselquist, Nielsen, & Hansson, 1998; Coyne & Orr, 2004; Price, 2008), or perhaps reinforced existing divergence (Grant & Grant, 1996; Hoskin, Higgie, McDonald, & Moritz, 2005; Lachlan & Servedio, 2004; Lynch & Baker, 1994; Mason et al., 2017). Relative to the accumulation of novel genetic markers or other traits, behavioral evolution via exchange of learned song can be rapid (Allender, Seehausen, Knight, Turner, & Maclean, 2003; Duckworth, 2009; West‐Eberhard, 1983). Further, species that learn their song evolve new dialects particularly quickly (Mason et al., 2017; but see Freeman, Montgomery, & Schluter, 2017). Rapid evolution of songs could mediate and/or supplement divergences across the Cochise Filter Barrier.

While we found evidence for a correlation between behavioral and genomic differentiation in this system, this does not rule out other mechanisms contributing to the divergence between the Sonoran and Chihuahuan populations. Ecological factors could have operated in tandem with behavioral factors in generating the divergence patterns seen. We dated the divergence between the Sonoran Desert and Chihuahuan Desert Northern Cardinals at approximately 990,000 years. This divergence is similar to estimates for bird species ages in the region, which typically date to the Pleistocene (Smith, Seeholzer, Harvey, Cuervo, & Brumfield, 2017). Divergence dates across the Cochise Filter Barrier, for various taxa, range from 500,000–5,000,000 years (Bryson, García‐Vázquez, & Riddle, 2011; Bryson, Jaeger, Lemos‐Espinal, & Lazcano, 2012; Klicka, Kus, & Burns, 2016; Leaché & Mulcahy, 2007; Myers et al., 2017; O'Connell, Streicher, Smith, & Fujita, 2017; Pyron & Burbrink, 2009; Smith & Klicka, 2013; Weyandt & Van Den Bussche, 2007; Wilson & Pitts, 2010; Zink & Blackwell‐Rago, 2000), suggesting that population connectivity and isolation have been dynamic in the region. For Northern Cardinals, ecological niche models (Smith et al., 2011) suggest that range shifts may have occurred during glacial cycling as the desert habitats expanded and contracted during the Pleistocene (Lang & Wolff, 2011; Mudelsee & Schulz, 1997). Differential gene flow rates between deserts may also be caused by differences in climatic suitability.

Demographic models show significantly higher support for a model with gene flow over a model of pure isolation, though the amount of gene flow was low. Our finding of minute mean gene flow since divergence is agnostic to the timing of gene flow itself, whether it was low and protracted or high and abbreviated. Gene flow rates in fastsimcoal2 are mean estimates over the entire period in which gene flow can occur, that is, from the time of divergence (or secondary contact) to present. As such, this does not capture temporal variation in gene flow rate. The failure to support a secondary contact model (Table A4) suggests that the gene flow happened earlier, rather than later, in the populations’ histories, and rejects the notion that contemporary introgression is occurring. Overall, the low amount of introgression indicates that while gene flow was possible across deserts, it was limited either in duration or in magnitude. This suggests that some isolating mechanism evolved between the populations diverging and completely cutting off gene flow before contemporary periods. Given our findings of rapid evolution of song discrimination within the Sonoran Desert (see below), it is likely that prezygotic isolation evolved early in the differentiation of the two desert groups, or was reinforced during this period of introgression.

Male Northern Cardinals, irrespective of desert, do not recognize Across‐Barrier dialects as conspecific. Under our tested hypothesis, this implies a pure isolation model of gene flow. Contrary to this, however, we found evidence for historical gene flow. This result is sensible if contemporary gene flow is nonexistent, as we assert above, and if song dialects evolve rapidly (e.g., West‐Eberhard, 1983; but see Noad, Cato, Bryden, Jenner, & Jenner, 2000 for song exchange without gene flow). The observation that Sonoran birds also do not recognize Distant songs as conspecific does not contradict this finding, though all explanations as to why this population shows such behavior are speculative. These findings may be due to differential gene flow between subpopulations (e.g., Grava et al., 2012; Lipshutz, 2017a; McDonald, Clay, Brumfield, & Braun, 2001; Robbins et al., 2014; Rosenfield & Kodric‐Brown, 2003; While et al., 2015).

The ability of the Sonoran population to discriminate from other Distant Sonoran songs reinforces the view that prezygotic isolation can evolve before genetic differentiation. The overall strength of song discrimination of Across‐Barrier dialects is consistent with reproductive isolation, and this type of finding is often used to delimit species based on the biological species concept (e.g., Caro, Caycedo‐Rosales, Bowie, Slabbekoorn, & Cadena, 2013; Cadena et al., 2015). However, we recommend caution, as even though it is defensible to assume that male response and female choice are tightly coupled, we did not test female choice. The few experiments done testing female choice in Northern Cardinals have not examined whether they discriminate against different genetic lineages (e.g., Yamaguchi, 1999), and so understanding their responses forms one of the critical next steps for this system. Anecdotally, during our experimental trials, female Northern Cardinals occasionally responded to playbacks (*N* = 9 trials). These females typically behaved similarly to the focal male of the trial and appeared more likely to investigate the speaker when hearing the Local dialect, rather than a novel dialect, tentatively suggesting that males and females behave similarly in this regard (K. L. Provost unpublished data).

All in all, the Cochise Filter Barrier structures Northern Cardinal populations both genetically, phenotypically, and behaviorally. Given our findings, the barrier appears to be facilitated, at least in part, by strong dialect differences that have evolved between the Sonoran and Chihuahuan deserts. These dialect differences affect song discrimination in male Northern Cardinals more potently than would be expected from geographic distance alone. Across the entire bird community, it is likely that different species have developed greater or fewer dialect differences across the barrier, which may be impacting the observed genetic semipermeability. We suggest that the song discrimination and genetic divergence we found in this species directly interact with each other to create the pattern of differentiation we see across the Cochise Filter Barrier. Future studies of birds codistributed across this barrier may find similar evidence for this pattern, and investigating many different mechanisms across multiple species at once would give insight into how the Cochise Filter Barrier, and other such barriers, worked to create the species diversity seen today.

## CONFLICT OF INTEREST

None declared.

## AUTHOR CONTRIBUTIONS

K.L.P. and B.T.S. conceived the study. K.L.P. and W.M.M. performed laboratory work. K.L.P. and B.T.S. performed behavioral work. K.L.P. ran analyses with input from other authors. K.L.P. drafted the manuscript. All authors edited the manuscript.

## DATA ACCESSIBILITY

Songs used to create treatments are have been uploaded to Xeno‐Canto (numbers XC434568‐XC434574 and XC434576‐XC434592). Sequences from ddRAD procedure have been uploaded to the NCBI Short Read Archive as submission SRP158705 and will be released upon publication. All data and scripts used to perform genetic and behavioral analyses are available on Dryad (awaiting curator revision, provisional https://doi.org/10.5061/dryad.fp4vv8s).

## Figures and Tables

**Figure A1:**
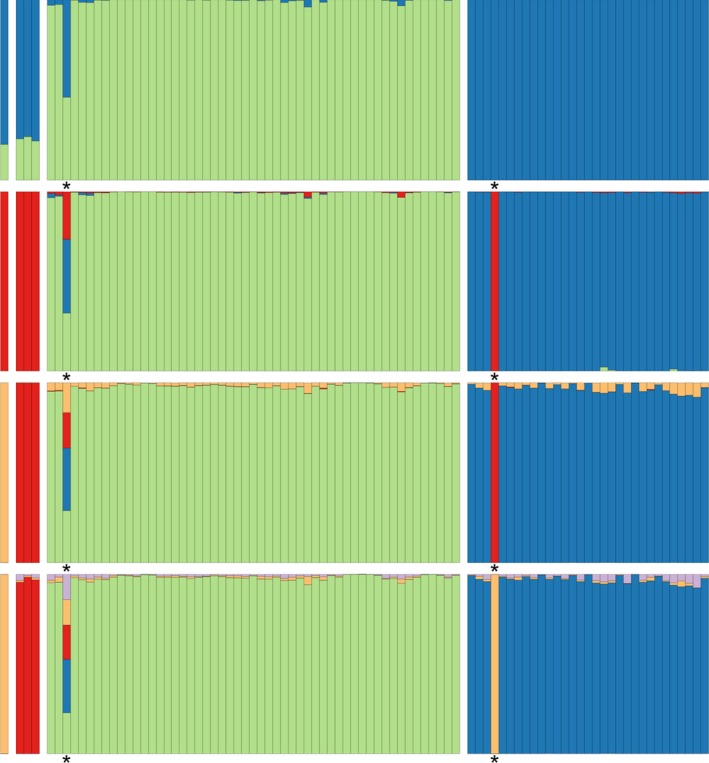
Outgroups, Sonoran Desert, and Chihuahuan Desert birds form distinct clusters. Results from STRUCTURE runs *K* = 2 (top) to *K* = 5 (bottom) are presented. Each vertical bar represents an individual bird, with the proportion of each color indicating assignment to that population. Groupings of bars separated by white gaps represent, from left, (a) *Cardinalis sinuatus*, (b) *C. cardinalis carneus*, (c) Sonoran *C. cardinalis*, and (d) Chihuahuan *C. cardinalis*. *Cardinalis sinuatus* is predominantly orange/red, *C. c. carneus* are predominantly red, Sonoran individuals are predominantly green, and Chihuahuan individuals are predominantly blue. Note that Chihuahuan group includes all samples east of the Cochise Filter Barrier, including individuals collected outside the Chihuahuan Desert proper. Dataset used is the outgroup‐included dataset, which retains two individuals with high missing data (indicated with asterisks). Order of bars is the same as in Main Text Figure 4, excepting outgroups and removed low‐data individuals

## APPENDIX 1

### GENETIC METHODS AND RESULTS

#### Sequence library preparation protocol

Double‐digest restriction‐site‐associated DNA sequence (ddRAD) libraries were prepared and sequenced at the University of Texas Austin Genomic Sequencing and Analysis Facility using a protocol modified from Peterson et al. (2012). In brief, the protocol is as follows: to 10 ng of genomic DNA add 0.1 μl each of restriction enzymes EcoRI and MspI and appropriate CutSmart buffers. Incubate at 37°C for 2 hr and then wash using 1.8× AMPure beads and 80% ethanol. Ligate P7 and P5 adapters for EcoRI and MspI for use in barcoding individuals as well as attaching later Illumina adapters. Wash again using 0.8× AMPure beads and 80% ethanol. Combine individuals into pools with unique adapters, 24 individuals per pool. Size select using a Pippin Prep and purify with a MinElute column. Use polymerase chain reaction settings as follows: denature at 98°C for 30 s and then begin 12 cycles of (a) 98°C for 10 s of denaturing, (b) 65°C for 30 s of annealing, and (c) 72°C for 30 s of extension. Finally, extend at 72°C for 5 min. Wash again using AMPure beads and 80% ethanol.

#### Bioinformatic processing of genomic reads

The raw genomic data were processed using PyRAD version 3.0.66 (Eaton, 2014). Settings were as follows: restriction site overhangs as AATTC for EcoRI and CGG for MspI; 16 processors; minimum read coverage for a cluster as six (such that any given individual needed to have at least six reads per locus); maximum number of sites with quality <20 of four; clustering threshold as 0.85 (such that loci needed to be 85% identical to be clustered as the same locus); data type as pairddrad; minimum samples in a final locus as four (such that only loci that had at least four individuals represented were reported); maximum individuals with a shared heterozygous site as three; maximum number of mismatches in barcodes as zero; filter out cut sites and adapters; maximum heterozygous sites in consensus sequences as 10; trim overhang on final loci as two on the left and two on the right.

We ran PyRAD with these settings twice: once with all individuals, and once excluding all four outgroup individuals of *C. sinuatus *and *C. c. carneus*. For the latter run, we also excluded one individual in which only three loci successfully sequenced. Only analyses using former run are reported in the main text; however, results from these two datasets did not differ substantially. In addition to having two datasets run independently in PyRAD, we also had no qualitative differences in results when we manually removed *C. sinuatus* and *C. c. carneus* from the outgroup‐included dataset and used that for analyses (results not shown).

#### Testing different thresholds for StructureSelector

We evaluated the best *K* value for our STRUCTURE runs using both the Evanno et al. (2005) method and the Puechmaille (2016) method, as implemented in StructureSelector (Li & Liu, 2018). For the Puechmaille (2016) method, we tested mean membership threshold values of 0.5, 0.6, 0.7, 0.8, and 0.9. We report only results from the 0.5 threshold in the main text.

#### Genomic structure results

From the dataset in which the outgroup was excluded, we collected 66,254 raw loci. After filtering for paralogs, this was reduced to 32,832 processed loci, from which we extracted 27,701 unlinked SNPs out of a total of 314,308 variable sites and 132,216 parsimony‐informative sites. Each individual had between 2,177 and 9,786 loci associated with it (mean ±standard deviation 7,058 ± 1,515, median 7,110). The individual with 2,177 loci was one of the individuals that was consistently mis‐assigned in STRUCTURE analyses (see below); all other individuals had at least 3,376 loci. The main text describes these results for dataset in which the outgroup was included. Differentiation between the Sonoran and Chihuahuan populations was relatively high. Nei’s *G*_ST_ was 0.33, while Hedrick’s *G*’_ST_ was 0.41.

As mentioned in the main text, two individuals had particularly high amounts of missing data (3 and 2,250 loci, or 99% and 92% missing, respectively, in the outgroup‐included dataset). We ran a preliminary STRUCTURE analysis including these individuals as well as with the outgroup taxa. The highest log‐likelihood support values for this run were for two (mean ± standard deviation −221,993 ± 81) and three (−197,641 ± 130) population clusters. Using the Puechmaille (2016) method, MedMed and MedMax always support *K* = 3 across all mean membership thresholds. However, MedMean and MaxMean support *K* = 3 for a threshold of 0.5, *K* = 2 for thresholds 0.6–0.7, and *K* = 1 for thresholds 0.8–0.9. Comparisons of ∆*K* (Evanno et al., 2005) showed highest support for *K* = 3, grouping *C. sinuatus and C. c. carneus* as one group separate from both the Sonoran and Chihuahuan individuals, which were clearly separated from each other except for the individuals with high missing data. The individual with 99% missing data was assigned with approximately equal probability to all groups irrespective of the analysis. The individual with 92% missing data was assigned to the Chihuahuan cluster for *K* = 2 and then assigned to outgroup clusters for *K* = 3 through *K* = 5 (Figure A1). Because of high missing data, we chose to remove these individuals from further analyses.

For the dataset described in the main text, with outgroups and individuals with high missing data removed, we tested five mean membership threshold values (Puechmaille, 2016) ranging from 0.5 to 0.9. Regardless of threshold, MedMed and MaxMed always support *K* = 2. MedMean and MaxMean also support *K* = 2 for thresholds 0.5–0.6, but for mean membership values 0.7–0.9 they support *K* = 1.

## APPENDIX 2 BEHAVIORAL METHODS AND RESULTS

### The role of male competition and female preference

Whether male song affects gene flow is contingent on the assumptions involving song, fitness, and female choice. If these assumptions bear out and individuals who do not match the nearby dialect are selected against, the song differences that have evolved between deserts may act as a potential reproductive barrier. One key assumption is that males and females have similar responses to dialect differences such that songs that males recognize as conspecific rivals would be attractive to females (Hunt et al., 2009; Uy et al., 2018). This requires that both sexes have the same discriminatory abilities and use the same mechanisms, which is not always the case (Lipshutz, 2017b). Females of different populations may prefer the same group of males in spite of divergent phenotypes (Baldassarre & Webster, 2013; Ryan & Rand, 1993; Coyne & Orr, 2004; Price, 2008). Similarly, for selection to occur against individuals that do not match the local dialect, females must not prefer rare males (Knoppien, 1985; Partridge, 1988). Captive‐raised Northern Cardinal females show no preference for familiar or novel conspecific songs, despite discriminating strongly against heterospecific songs (Yamaguchi, 1999), but it is unclear how females regard individuals from highly diverged genetic lineages of the same species. In this study, males treat songs from across the Cochise Filter Barrier as heterospecific, and given evidence that females are more discriminatory than males (Uy et al., 2018), it is likely that females would behave similarly. Anecdotally, females observed while performing playback experiments appeared more likely to investigate the speaker when hearing the Local dialect, rather than a novel dialect, suggesting that males and females behave similarly in this regard (K. L. Provost unpublished data).

Either scenario (differences in discriminatory ability between the sexes or female preference for novel males) could lead to a disconnect between behavior and fitness if female choice is the dominant method of sexual selection. There is evidence that differences in male–male competition can impact fitness without being directly mediated by female choice, but this evidence has proven to be troublesome to interpret. Males on high‐quality territories have higher breeding success (Wolfenbarger, 1999a, 1999b), but the link between songs and territories appears convoluted. Simple, short song structures were found in Northern Cardinal males on high‐quality territories (Conner et al., 1986; Narango & Rodewald, 2015, 2018), but these same song qualities may also lead to delayed mating success (Ritchison, 1988) and appear to be heavily influenced by the urbanization of habitat and density of individuals (Narango & Rodewald, 2015, 2018). There may also be fitness consequences to songs learned mediated by parasites, as found in other species, which could reflect trade‐offs between male competitive ability and ecological adaptation (MacDougall‐Shackleton, Derryberry, & Hahn, 2002; Qvarnström, Vallin, & Rudh, 2012); however, there is no current evidence that songs and parasite loads are directly linked in Northern Cardinals. Rather than impacts on territory quality, song structure’s impacts on song matching between individuals may be more important for this species. Female Northern Cardinals that match the songs of their mates decrease nestling provisioning by the male, while singing a different song has the opposite effect (Halkin, 1997). Song matching happens frequently between neighboring males, apparently even more so during territorial disputes (Lemon, 1968). In either scenario, whether song structure is linked to territory quality, defense, or nest provisioning, it appears likely that vagrant males should encounter difficulties in forming and maintaining territories (Searcy & Nowicki, 2005) and providing for their mates and offspring effectively.

### Playback experiment protocol details

Sonoran Desert playback treatments comprised *C. cardinalis* songs recorded in one of three localities. Songs from Portal, AZ, served as the “Local” treatment, as they came from the Sonoran population whose responses we measured. Songs from western Arizona (Bill Williams River, AZ, Santa Maria River, AZ, and Wenden, AZ) served as the “Distant” treatment, as they were from a population within the same desert as the Local population. These Distant localities are ~35–50 km apart from each other, with Santa Maria River being ~490 km from Portal, AZ, Bill Williams River being ~530 km, and Wenden being ~460 km. Songs from Rattlesnake Springs, NM, served as an “Across‐Barrier” treatment and represented both a different genetic population and a large geographic separation from the Local population (approximately ~450 km away and within the Chihuahuan Desert).

Chihuahuan Desert experiment treatments were similar, in that there were Local, Distant, and Across‐Barrier songs. However, the songs themselves were different within these categories. The “Local” songs for the Chihuahuan Desert side were recorded in Big Bend National Park, TX. Note that the easternmost and westernmost localities in Big Bend National Park are ~65 km apart due to scarcity of suitable *C. cardinalis* habitat. “Distant” songs for the Chihuahuan Desert were recorded in eastern Texas at Warbler Vista, Balcones Canyonlands National Preserve, TX, as well as Austin, TX. These two localities were ~40 km apart, and Austin is ~520 km from the most eastern Big Bend National Park site. The “Across‐Barrier” songs for the Chihuahuan Desert were the Portal, AZ, songs, identical to the Sonoran Local songs. Again, these represent a different genetic population and a large geographic separation (approximately ~620 km from the most western Big Bend National Park site).

Because of the large geographic separation, Distant songs and Across‐Barrier songs should have been novel to the Local population for both desert populations (Lemon, 1975). We chose these localities to compare the effects of both distance and presumed genetic relatedness on a population’s response to a recording. Comparing the Local and Distant treatments allowed us to assess the impact of distance while controlling for genetic relatedness, as there is no significant mitochondrial or nuclear (Smith & Klicka, 2013; Smith et al., 2011) phylogeographic structure within each desert. Comparing the Distant and Across‐Barrier treatments allowed us to assess the impact of genetic relatedness while controlling for distance. We also used a Cactus Wren (*Campylorhynchus brunneicapillus*) recording from Portal, AZ, as a heterospecific Control treatment for both populations. We assumed that between‐desert differences in Cactus Wren songs would not affect the response of Northern Cardinal males to those songs. This recording controlled for differences in aggression relating to the experimental setup, in particular the presence of humans and the potential for birds to investigate (or be scared off by) sudden loud noises on their territories.

To select the songs we used for the treatments, we chose recordings in which the male *C. cardinalis* was prominently singing and background noise was low. Prior to processing, we acquired 24 Portal, AZ, songs, 12 Rattlesnake Springs, NM, songs, seven western Arizona songs, 15 Big Bend National Park, TX, songs, and 10 eastern Texas songs. We edited songs using Audacity 2.0.6 (Audacity Team, 2014) to normalize recording amplitude, remove remaining background noise, and cut sections of recordings with potentially disruptive sound such as human voice or instances of duetting. We also converted all songs from stereo to mono tracks if needed. Additionally, for the Chihuahuan treatments, songs were compressed to increase volume using Audacity’s “compressor” function (−60 db, −40 db, 2:1, 0.2 s, 1.0 s, makeup gain checked, compress based on peaks checked).

Raw recordings contained an average of 8.19 clear songs uninterrupted by a disruptive sound (range: 1–17). We linked songs from a given area into a stimulus set of approximately 3 min duration (range: 2:59–3:10 min). The stimulus sets contained an average of 20.31 songs (range: 15–33) for an average rate of 6.77 songs per minute (range: 5–11). Each stimulus set included three 50‐s bouts of song, with 10‐s periods of silence following each bout. We added these silent periods to mimic multiple singing bouts, rather than one continuous bout. Northern Cardinals in these areas rarely sang continuously for 3 min (K. L. Provost, & B. T. Smith personal observation) and we wanted to mimic a natural encounter as closely as possible. After processing, we had five stimulus sets for each treatment except for the Rattlesnake Springs, NM, and Control treatments. We processed seven Rattlesnake Springs, NM, and one Control treatment stimulus set.

### Behavioral study site details

To avoid double sampling, we set up sites as proxies for individual males. Northern Cardinals of both sexes are fiercely territorial toward intruders and their territories are stable through the breeding season (Gould, 1961); therefore, major territorial shifts were not expected during the trials and we assumed that individuals found on territories were the territory holders. Sites were geographically spaced apart using a Garmin GPSMAP 62s to minimize territorial overlap and selected by placing GPS points in habitat known to have territorial male Northern Cardinals. Distances between Sonoran nearest‐neighbor sites ranged from 38 to 205 m, with the maximum distance between two sites being 4.12 km. Distances between Chihuahuan nearest‐neighbors were 92 to 204 m. Patches of suitable Northern Cardinal habitat were small and widely separated in the Chihuahuan Desert, and so the maximum distance between two sites was 67.59 km. Sonoran sites were linked together as seven transects (length range: 1,804–4,067 m) of 6–12 points whose playbacks we always completed in the same order (with the exception of two sites on 2 days that were interrupted by equipment failure and dangerous conditions, respectively). Chihuahuan sites were linked together as six transects (length: 1,043–26,374 m) of 7–13 points. These were also completed in the same order, but dangerous conditions led to 7 days in which some points were skipped.

We performed playbacks on only one transect per day. Sites within transects were at least 100 m apart to further minimize repeat testing of the same territories, with one exception (two Chihuahuan points 92 m apart). As such, nearest‐neighbor sites were not necessarily included on the same transect. All Sonoran sites were located between 1,408 and 1,535 m above sea level, and Chihuahuan sites between 559 and 1,108 m above sea level. Playback trials proceeded from 16 May to 16 June 2015 between 5:10 a.m. and 9:20 a.m. local time for Sonoran birds and from 4 June to 1 July 2016 between 6:31 a.m. and 10:59 a.m. local time for Chihuahuan birds, with daily trials beginning just prior to sunrise. We performed all playbacks at each site at nearly the same time each day (range of time differences: 2:50 min to 1 hr 39:20 min) and never conducted more than one playback per site per day with two exceptions: Two Chihuahuan points had multiple playbacks and one of these same points had a total time difference of 3 hr 14:50 min. We completed all trials within 7 days of initiating experiments at a site, with the exception of one trial that took 8 days and one that took nine. Males had at least approximately 24 hr between treatments (Sonoran median 23.9 hr, range 23.4–72.3 hr; Chihuahuan median 24.1 hr, range 22.3–48.5 hr) to return to a non‐disturbed state, with the exception of the previously mentioned points (range 0.2–98.9 hr).

### Playback protocol details

We conducted four playbacks at each site, randomly selecting one stimulus set from each of the four treatment localities (Local, Distant, Across‐Barrier, and Control). We randomized the order of the treatments at each site prior to beginning any playbacks. Post hoc, we tested the impact of individuals potentially hearing themselves or their neighbors (e.g., Brooks & Falls, 1975a; 1975b; Falls & Brooks, 1975). To do this, we excluded all sites that were within 200 m of a recording if those sites also heard that recording. This removed 12 sites from the Sonoran dataset and two sites from the Chihuahuan dataset. However, this did not qualitatively change the results of any analysis so we do not present those results. We used an HTC 6500LVW cell phone running Android version 5.0.2 and an HMDX Jam Bluetooth speaker (model HX‐P230GRA) with a ~1‐m‐long 3.5‐mm audio cable to play the recordings. We placed the speaker on a tripod ~1 m off the ground.

Of the 512 total trials, 19 pulled in multiple male *C. cardinalis* (10 Sonoran, 9 Chihuahuan). For these 19 trials, we calculated the aggression values (see Main Text) by adding Flybys, Close Songs, and Far Songs from each male, by considering alarm calls from either male as indicating Calls, and by calculating the minimum Distance by Time for each 10 s interval. In doing this, we assumed that both males were directing their aggression toward the speaker. We also ran a dataset in which any site which had multiple males was excluded. This did not qualitatively affect the results of the Sonoran dataset. For the Chihuahuan dataset, the only change is that individuals are now significantly more aggressive to Across‐Barrier songs than to Control songs (see Main Text). Males hearing Across‐Barrier songs are still significantly less aggressive than males hearing Distant and Local songs. We do not believe that this substantially impacts our conclusions. In addition, some trials attracted female *C. cardinalis*, juvenile *C. cardinalis*, and adult *C. sinuatus* of both sexes. We assumed the presence of these individuals would not affect the response of *C. cardinalis* males to the playback.

### Analysis of behavioral data details

We compared the responses to the four treatments to determine how Northern Cardinals respond to Local and Distant populations across biogeographic barriers. The five aggression measures were reduced to a single composite aggression measure via PCA using the prcomp function in the built‐in package stats 3.1.1 in R version 3.1.1 (R Core Team, 2014). We calculated the principal components using Response period (Playback and Post‐Playback combined) data. Data from the Chihuahuan and Sonoran experiments were processed both separately and together, giving three sets of principal components. The first principal component (PC1) explained 58.2% of the variation in the Sonoran‐only Response period data, 52.4% of variation in the Chihuahuan‐only Response period, and 54.6% of the variation when both deserts were combined. We used the resulting loadings to calculate a posteriori the first principal component for the Pre‐Playback period, or “Pre‐Treatment Aggression” measure. This measure served as a control for aggression present at a site before the recording began playing, which could influence the final aggression levels. The combined‐deserts PC1 and Pre‐Treatment Aggression measures were used only to assess whether there were differences between the experiments in total aggression levels. To accomplish this, we performed a two‐factor analysis of variance using the aov function in the stats package in R, setting up our models as follows: PC1 (or Pre‐Treatment Aggression) ~ Treatment + Population Tested + Treatment: Population Tested, with the final term in the model representing the interaction between those two variables.

We used generalized linear mixed models (GLMMs) to determine whether there was a correlation between aggression (PC1) and treatment. PC1 was the dependent variable in these models, with stimulus set and the Pre‐Playback control variable Pre‐Treatment Aggression as fixed effects. We included the random effects of site and stimulus set in our models to control for individual site differences and differential responses to the various stimulus sets from the same locality. GLMMs were run using the Sonoran‐only PCA and the Chihuahuan‐only PCA data, not the combined‐deserts PCA data.

To evaluate the importance of treatment locality as a predictor, we compared the outputs of: (a) a model containing all variables and (b) a model will all variables except treatment. We assessed model fit using AIC_C_ as calculated by the AICcmodavg version 2.0‐4 (Mazerolle, 2015) and qpcR version 1.4‐0 (Spiess, 2014) R packages. We chose the model with the smallest AIC_C_ value (as measured by ∆AIC_C_) as the best model. We set significance level (alpha level) to 0.05. We evaluated the significance of treatment and Pre‐Treatment Aggression predictors using a Wald chi‐square test with the function ANOVA in the package car version 2.1‐3 in R (Fox & Weisberg, 2011).

We also assessed model fit by calculating *R* ^2^ values (as calculated by *R* ^2^ = 1 − (*SSres*/*SStot*), where *SSres *indicates the sum of squares of the residuals as given by the GLMM, and *SStot *indicates the sum of squares of the actual aggression values). To compensate for the influence of the number of predictors on *R* ^2^ values (Fisher, 1924), we also calculated adjusted *R* ^2^ (adj*R* ^2^, as calculated by adj*R* ^2^ = 1 − (1 − *R* ^2^)*((*n *− 1)/(*n*− *p*− 1)), where *n* is the number of observations and *p* is the number of predictor variables).

We generated GLMMs using the glmer function in the R package lme4 version 1.1‐10 (Bates, Mächler, Bolker, & Walker, 2015) with Gamma error structure and log‐link as detected by the qqp function in the package MASS version 7.3‐33 (Venables & Ripley, 2002). When assessing Gamma error structure, the qqp function requires all response values to be non‐negative, so we adjusted PC1 and Pre‐Treatment Aggression accordingly. For the Sonoran data, we adjusted PC1 to have a mean of 2.0 rather than a mean of 0.0 by adding 2.0 to each value. For the Chihuahuan data, we adjusted PC1 to have a mean of 1.0 rather than 0.0 by adding 1.0 to each value. In addition, Sonoran PC1 was positively correlated with aggression while Chihuahuan PC1 was negatively correlated with aggression—as such, we multiplied the Chihuahuan data by −1.0 to run in the GLMMs. We compared the results from two different optimizer algorithms BOBYQA (Powell, 2009) and L‐BFGS‐B (Byrd, Lu, Nocedal, & Zhu, 1995) using the R package optimx version 2013.8.7 (Nash, 2014; Nash & Varadhan, 2011). However, both optimization algorithms gave similar parameters for all four datasets, so we only report results from the BOBYQA algorithm.

### Testing sensitivity of analyses to failed detections

As mentioned in the main text, we failed to detect males at some sites, which we defined as never detecting a male within our study site either by sight or by sound. We analyzed our resulting data both with and without these sites to see whether it impacted our results. Here, we present the results from those analyses alongside the results from the main text.

For the Sonoran data, the GLMM including Treatment was a better fit to the aggression data than the model without Treatment (∆AIC_C_ = 11.38), though both models had equivalent adjusted *R* ^2^ values (0.90). Wald tests indicated that both Treatment and Pre‐Treatment Aggression were significant factors in the full model (Treatment: *χ* ^2^ = 53.07, *df* = 3, *p*‐value = 1.52 × 10^−12^; Pre‐Treatment Aggression: *χ* ^2^ = 31.89, *df* = 1, *p*‐value = 1.63 × 10^−8^). Specifically, Sonoran male Northern Cardinals were significantly more aggressive to Local stimulus sets than to those from any other location (all *p*‐values <= 3.02 × 10^−10^). By contrast, there were no significant differences in aggression across Distant, Across‐Barrier, or Control stimulus sets (all *p*‐values >= 0.354). Excluding sites without detections had no qualitative bearing on these results, though it slightly reduced the fit of the model (∆AIC_C_ = 9.97, adjusted *R* ^2^ values range: 0.89–0.90, Treatment *p*‐value = 1.00 × 10^−10^).

For the Chihuahuan data, both models had equal adjusted *R* ^2^ values (0.64). While AIC_C_ scores supported the GLMM including Treatment over the model without Treatment, the difference was not significant (∆AIC_C_ = 1.22 for both). Nevertheless, Wald tests indicated that both Treatment and Pre‐Treatment Aggression were significant factors in the full model (Treatment: *χ* ^2^ = 9.22, *df* = 3, *p*‐value = 0.027; Pre‐Treatment Aggression: *χ* ^2^ = 55.86, *df* = 1, *p*‐value = 7.77 × 10^−14^). Specifically, Chihuahuan male Northern Cardinals were significantly more aggressive to Local and Distant stimulus sets than to Across‐Barrier or Control stimulus sets (all *p*‐values <= 0.041). Local and Distant were not statistically different, and neither were Across‐Barrier and Control (all *p*‐values > = 0.539). When sites without detections were excluded, the GLMM with treatment was significantly supported over the model without (∆AIC_C_ = 4.79), though the model without treatment had greater explanatory power according to adjusted *R* ^2^ (Null = 0.71, Full = 0.67). As before, both Treatment and Pre‐Treatment Aggression were significant factors in the full model (Treatment: *χ* ^2^ = 20.79, *df* = 3, *p*‐value = 1.16 × 10^−4^; Pre‐Treatment Aggression: *χ* ^2^ = 33.71, *df* = 1, *p*‐value = 6.39 × 10^−9^) and Chihuahuan males were significantly more aggressive to Local and Distant stimulus sets than to other datasets.

**Table A1:** Catalog numbers of specimens used for genomic analyses

Genus	Species	Specimen	Genus	Species	Specimen
*Cardinalis*	*cardinalis*	AMNH:Birds:DOT−8616	*Cardinalis*	*cardinalis*	UWBM:Bird:101030
*Cardinalis*	*cardinalis*	AMNH:Birds:DOT−4953	*Cardinalis*	*cardinalis*	UWBM:Bird:101158
*Cardinalis*	*cardinalis*	AMNH:Birds:DOT−4954	*Cardinalis*	*cardinalis*	UWBM:Bird:101159
*Cardinalis*	*cardinalis*	AMNH:Birds:DOT−4955	*Cardinalis*	*cardinalis*	UWBM:Bird:101160
*Cardinalis*	*cardinalis*	MSB:Bird:18505	*Cardinalis*	*cardinalis*	UWBM:Bird:103345
*Cardinalis*	*cardinalis*	MSB:Bird:22180	*Cardinalis*	*cardinalis*	UWBM:Bird:111638
*Cardinalis*	*cardinalis*	MSB:Bird:23112	*Cardinalis*	*cardinalis*	UWBM:Bird:77856
*Cardinalis*	*cardinalis*	MSB:Bird:24193	*Cardinalis*	*cardinalis*	UWBM:Bird:77867
*Cardinalis*	*cardinalis*	MSB:Bird:24397	*Cardinalis*	*cardinalis*	UWBM:Bird:77974
*Cardinalis*	*cardinalis*	MSB:Bird:24474	*Cardinalis*	*cardinalis*	UWBM:Bird:77978
*Cardinalis*	*cardinalis*	MSB:Bird:24478	*Cardinalis*	*cardinalis*	UWBM:Bird:78054
*Cardinalis*	*cardinalis*	MSB:Bird:40218	*Cardinalis*	*cardinalis*	UWBM:Bird:81257
*Cardinalis*	*cardinalis*	MSB:Bird:44288	*Cardinalis*	*cardinalis*	UWBM:Bird:81260
*Cardinalis*	*cardinalis*	MSB:Bird:44289	*Cardinalis*	*cardinalis*	UWBM:Bird:81262
*Cardinalis*	*cardinalis*	MSB:Bird:44290	*Cardinalis*	*cardinalis*	UWBM:Bird:82414
*Cardinalis*	*cardinalis*	TCWC:Birds:14606	*Cardinalis*	*cardinalis*	UWBM:Bird:82417
*Cardinalis*	*cardinalis*	TCWC:Birds:14644	*Cardinalis*	*cardinalis*	UWBM:Bird:82421
*Cardinalis*	*cardinalis*	TCWC:Birds:14696	*Cardinalis*	*cardinalis*	UWBM:Bird:82428
*Cardinalis*	*cardinalis*	TCWC:Birds:15429	*Cardinalis*	*cardinalis*	UWBM:Bird:82433
*Cardinalis*	*cardinalis*	TCWC:Birds:15895	*Cardinalis*	*cardinalis*	UWBM:Bird:82454
*Cardinalis*	*cardinalis*	TCWC:Birds:16184	*Cardinalis*	*cardinalis*	UWBM:Bird:82720
*Cardinalis*	*cardinalis*	TCWC:Birds:16185	*Cardinalis*	*cardinalis*	UWBM:Bird:82721
*Cardinalis*	*cardinalis*	TCWC:Birds:16186	*Cardinalis*	*cardinalis*	UWBM:Bird:82724
*Cardinalis*	*cardinalis*	TCWC:Birds:16188	*Cardinalis*	*cardinalis*	UWBM:Bird:82745
*Cardinalis*	*cardinalis*	TCWC:Birds:16196	*Cardinalis*	*cardinalis*	UWBM:Bird:83937
*Cardinalis*	*cardinalis*	TCWC:Birds:16276	*Cardinalis*	*cardinalis*	UWBM:Bird:83942
*Cardinalis*	*cardinalis*	TCWC:Birds:16440	*Cardinalis*	*cardinalis*	UWBM:Bird:83949
*Cardinalis*	*cardinalis*	TCWC:Birds:16488	*Cardinalis*	*cardinalis*	UWBM:Bird:83990
*Cardinalis*	*cardinalis*	TCWC:Birds:16681	*Cardinalis*	*cardinalis*	UWBM:Bird:84009
*Cardinalis*	*cardinalis*	TCWC:Birds:16764	*Cardinalis*	*cardinalis*	UWBM:Bird:84029
*Cardinalis*	*cardinalis*	TCWC:Birds:16765	*Cardinalis*	*cardinalis*	UWBM:Bird:84077
*Cardinalis*	*cardinalis*	TCWC:Birds:16766	*Cardinalis*	*cardinalis*	UWBM:Bird:86460
*Cardinalis*	*cardinalis*	TCWC:Birds:17027	*Cardinalis*	*cardinalis*	UWBM:Bird:86475
*Cardinalis*	*cardinalis*	TCWC:Birds:17028	*Cardinalis*	*cardinalis*	UWBM:Bird:86541
*Cardinalis*	*cardinalis*	TCWC:Birds:17152	*Cardinalis*	*cardinalis*	UWBM:Bird:86559
*Cardinalis*	*cardinalis*	TCWC:Birds:17198	*Cardinalis*	*cardinalis*	UWBM:Bird:89013
*Cardinalis*	*cardinalis*	TCWC:Birds:17199	*Cardinalis*	*cardinalis*	UWBM:Bird:89016
*Cardinalis*	*cardinalis*	TCWC:Birds:17226	*Cardinalis*	*cardinalis*	UWBM:Bird:89068
*Cardinalis*	*cardinalis*	TCWC:Birds:17242	*Cardinalis*	*cardinalis*	UWBM:Bird:90704
*Cardinalis*	*sinuatus*	UWBM:Bird:100163	*Cardinalis*	*cardinalis*	UWBM:Bird:90705
*Cardinalis*	*cardinalis*	UWBM:Bird:100619	*Cardinalis*	*cardinalis*	UWBM:Bird:90800
*Cardinalis*	*cardinalis*	UWBM:Bird:100620	*Cardinalis*	*cardinalis*	UWBM:Bird:90817
*Cardinalis*	*cardinalis*	UWBM:Bird:100621	*Cardinalis*	*cardinalis*	UWBM:Bird:90857
*Cardinalis*	*cardinalis*	UWBM:Bird:100622	*Cardinalis*	*cardinalis*	UWBM:Bird:90864
*Cardinalis*	*cardinalis*	UWBM:Bird:100623			

Notes

AMNH: American Museum of Natural History; MSB: Museum of Southwestern Biology; TCWC: Texas A&M Biodiversity Research and Teaching Collections; UWBM: University of Washington Burke Museum of Natural History.

**Table A2:** Playback experiment transect data for the Sonoran Desert

Sonoran Desert		Songs played (Songs available)		
Transect	Sites	Local: Portal, AZ	Distant: Western Arizona	Across‐Barrier: Rattlesnake Springs, NM
Son.A	8	5 (5)	5 (5)	6 (7)
Son.B	11	5 (5)	5 (5)	3 (7)
Son.C	10	5 (5)	5 (5)	5 (7)
Son.D	12	4 (5)	5 (5)	5 (7)
Son.E	10	4 (5)	5 (5)	5 (7)
Son.F	10	3 (5)	4 (5)	5 (7)
Son.G	6	3 (5)	3 (5)	4 (7)

Notes

Transect gives the name of the transect (for instance, Son.A would indicate Transect A in the Sonoran Desert, and Chi.B would indicate Transect B in the Chihuahuan Desert). Sites indicates how many individual sites were included in the transect. Local, Distant, and Across‐Barrier show the number of songs played (out of total number of songs available) to represent the associated locality across the entire transect (see Figure 5 for localities). All sites heard the same Control song.

**Table A3:** Playback experiment transect data for Chihuahuan Desert

Chihuahuan Desert		Songs played (Songs available)		
Transect	Sites	Local: Big Bend, TX	Distant: Eastern Texas	Across‐Barrier: Portal, AZ
Chi.A	13	5 (5)	5 (5)	5 (5)
Chi.B	8	5 (5)	4 (5)	5 (5)
Chi.C	7	4 (5)	4 (5)	3 (5)
Chi.D	11	4 (5)	5 (5)	4 (5)
Chi.E	11	4 (5)	4 (5)	4 (5)
Chi.F	11	5 (5)	4 (5)	5 (5)

Notes

Column headings are as in Table A2.

**Table A4:** Model selection results for fastsimcoal2 demographic analyses

Model	AIC (∆AIC)	Div. time (Sec. Con.)	Sonoran *N*_e_	Chihuahuan *N*_e_	Migration rate
Asymmetric migration	12,876.3 (0)	704,830	56,080	1,126,195	Son→Chi: 1.60 × 10^−^^6^ Chi→Son: 1.78 × 10^−^^6^
Migration Son→Chi	12,880.0 (3.7)	727,197	230,555	1,132,828	Son→Chi: 1.67 × 10^−^^6^ Chi→Son: N/A
Symmetrical migration	12,880.6 (4.3)	648,613	62,051	1,131,211	Son→Chi: 1.40 × 10^−^^6^ Chi→Son: 1.40 × 10^−^^6^
Secondary contact	12,940.9 (64.5)	509,117 (18,698)	96,675	1,125,978	Son→Chi: 1.34 × 10^−^^6^ Chi→Son: 1.78 × 10^−^^6^
Migration Chi→Son	13,345.7 (469.3)	505,802	61,261	1,133,518	Son→Chi: N/A Chi→Son: 2.30 × 10^−^^6^
Pure isolation	14,005.7 (1,129.3)	509,464	258,711	1,134,865	Son→Chi: N/A Chi→Son: N/A

Notes

See Figure A2 for graphical representations of models. Table shows results from highest maximum likelihood run for each model. For other results, see data on Dryad.

Div. Time: Divergence time between Sonoran and Chihuahuan desert populations; Sec. Con: secondary contact time between Sonoran and Chihuahuan populations.
